# Incidence of and County Variation in Fall Injuries in US Residents Aged 65 Years or Older, 2016-2019

**DOI:** 10.1001/jamanetworkopen.2021.48007

**Published:** 2022-02-11

**Authors:** Geoffrey Hoffman, Nils Franco, Jennifer Perloff, Joanne Lynn, Safiyyah Okoye, Lillian Min

**Affiliations:** 1 Department of Systems, Populations, and Leadership, University of Michigan School of Nursing, Ann Arbor, Michigan; 2 ATI Advisory, Washington, DC; 3 Brandeis University, Waltham, Massachusetts; 4 Institute for Accountable Care, Washington, DC; 5 Altarum, Washington, DC; 6 Johns Hopkins School of Nursing, Baltimore, Maryland; 7 Division of Geriatric and Palliative Medicine, University of Michigan, Ann Arbor, Michigan; 8 VA Ann Arbor Healthcare System, Geriatric Research Education and Clinical Center, Ann Arbor, Michigan

## Abstract

This cross-sectional study examines national trends and geographic variation of fall injury rates among older US residents.

## Introduction

Falls cause widespread disability, death, and substantial health care spending; hip fractures and other injuries from falls affect 4.5 million older US residents annually and cost Medicare $15 to $30 billion annually.^1^ Prior reports of increased fall injury incidence trends are limited by use of self-reported survey data, which severely undercount fall injuries.^2,3^ We sought to document national trends and geographic variation in fall injuries.

## Methods

The University of Michigan institutional review board deemed this cross-sectional study exempt from review and granted waiver of informed consent because it used existing, deidentified data. This study followed the Strengthening the Reporting of Observational Studies in Epidemiology (STROBE) reporting guideline.

We conducted a retrospective observational analysis using 100% Medicare Part A and B claims from January 1, 2016, through December 31, 2019, including beneficiaries with continuous Parts A and B coverage during each analytic year. We restricted the population to older beneficiaries (aged at least 65 years), given their heightened fall risk. Race and ethnicity data were derived using the Research Triangle Institute race code variable from the Medicare Master Beneficiary Summary File. We applied an algorithm^4^ (updated to *International Statistical Classification of Diseases and Related Health Problems, Tenth Revision [ICD-10]*) that captures moderate to severe injuries based on the mechanism (the external cause of morbidity and mortality codes) or diagnosis (hospital, emergency department, or outpatient claims) of injury, which had 83% positive predictive value in a prior validation study.^4^ Injuries included fractures, wounds, dislocations, and contusions. The identification of injury episodes accounted for multiple treatments for a single fall injury, and distinguished multiple injuries per individual (as applicable). We included fatal injuries.

Quarterly fall injury rates equal the sum of the fall injuries identified, divided by the sum of analyzed person-quarters in that quarter, multiplied by 100 000. Rates were age and sex adjusted. We calculated the mean annual growth rate and overall rate increase. Counties with fewer than 11 fall injuries in a quarter were omitted for that quarter for all analyses. Statistical analysis was performed using SAS software version 9.4 (SAS Institute) and R version 4.1.0 (R Project for Statistical Computing) from June to December 2021.

## Results

Of 120.7 million claims identified from 2016 to 2019, 67.4 million (55.8%) involved women, 2.4 million (7.9%) involved Black adults, 1.7 million (4.8%) involved Hispanic adults, 24.3 million (80.9%) involved non-Hispanic White adults, 66.9 million (55.4%) involved adults aged 65 to 74 years, 36.2 million (30.0%) involved adults aged 75 to 84 years, and 17.6 million (14.6%) involved adults aged at least 85 years. The mean (SD) age-adjusted and sex-adjusted national quarterly fall injury rate increased from 1332 (310) fall injuries per 100 000 persons to 1391 (305) fall injuries per 100 000 persons, a 4.4% overall increase (or 1.5% annual growth) (Table). Counties at the 10th percentile of fall rates had a 42.9% lower fall injury compared with the 90th percentile (Figure).

**Table.** Fall Injuries per 100 000 Person-Quarters: Annual Average and Overall Increase

County percentile, mean	Quarterly rates					%	
	2016	2017	2018	2019	2016 -2019	Average annual increase	Overall increase
90th	1987	1982	2036	2098	2026	1.8	5.6
Median	1512	1536	1566	1580	1550	1.5	4.5
10th	1117	1151	1178	1189	1157	2.1	6.4

Author calculations of age-adjusted and sex-adjusted quarterly rates per 100 000 persons using 2016 to 2019 Medicare data. Each period’s value is the sum of the fall injuries identified divided by the sum of analyzed person-quarters in that period, multiplied by 100 000. The average annual growth rate, which is the rate of change each year that would result in the overall increase, is equal to the 2019 value divided by the 2016 value, to the power of 1/3, minus 1. The overall increase is the 2019 value divided by the 2016 value, minus 1. If a county had fewer than 11 fall injuries in a quarter, that county-quarter is omitted from the numerator and denominator for the period analyzed. For years 2016 to 2019, this resulted in 2912, 2909, 2916, and 2932 counties with at least 1 valid quarter of data and 11 046, 11 076, 11 170, and 11 209 valid county quarters each year, respectively.

**Figure.**
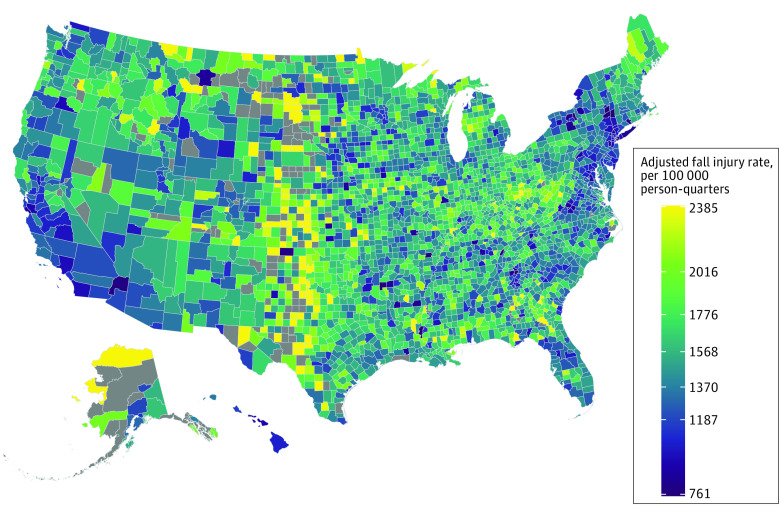
Map of US Counties With Fall Injury Rates Per 100 000 Person-Quarters, 2016-2019 Author calculations of age and sex-adjusted quarterly rates per 100 000 persons using 2016 to 2019 100% Medicare Parts A and B data. Each county’s value is the sum of identified fall injuries divided by the sum of analyzed person-quarters among county residents during 2016 through 2019. Quarterly data from counties with fewer than 11 fall injuries are omitted from the numerator and denominator for that county. Legend ticks represent the distribution of the cross-quarter county rates: from top to bottom, the upper outlier bound (Q_3_ + 1.5 × IQR), the 90th percentile, the third quartile (Q_3_), the median, Q_1_, the 10th percentile, and the lower outlier bound (Q_1_−1.5 × IQR). Counties with no uncensored quarterly data (where all quarters had fewer than 11 fall injuries) are gray.

## Discussion

In this pooled cross-sectional study of US fall injuries, incidence continued to increase at 1.5% per year, consistent with earlier trends.^2^ This translates to an additional 106 000 new fall injuries nationally, or more than $1 billion in fall injury spending during the study period.^1,5^ Substantial county-level variability in falls suggests modifiable population-level risk factors that may include physical activity levels, the built environment, prescribing practices, and fall prevention program availability and use.

Although fall prevention programs commonly target severe injuries, moderate injuries, including many that do not require medical treatment, may result in critical downstream health impacts that include fear of falling, social isolation, and declines in mobility and physical conditioning.^6^ These national data, which include severe and moderate injuries, may help planners identify risks, although improvement efforts should also target noninjurious falls.

This study is subject to several limitations. First, we excluded individuals enrolled in Medicare Advantage, which limits generalizability of our findings. Also, we only capture falls requiring medical care, likely making these estimates conservative. Furthermore, claims-based measures that rely on diagnostic data may be more sensitive in areas with greater coding intensity.

Our findings present population-targeted risk management opportunities to stem a growing and costly public health challenge. As injuries increase, improvements in policies and practices to prevent falls seem both possible and prudent.
